# Primary Psoas Abscess in a Pediatric Patient: A Case Report

**DOI:** 10.7759/cureus.26206

**Published:** 2022-06-22

**Authors:** Isra Idris, Maysam Aburas, Fernanda Ibarra Martinez, Elizabeth Osei-Kuffuor, Kayla Adams, Taurah Dizadare, Marsha Medows

**Affiliations:** 1 Pediatrics, Woodhull Medical Center, New York City, USA; 2 Internal Medicine, King Fahad Medical City, Riyadh, SAU; 3 Pediatrics, St. George's University, New York City, USA; 4 Pediatrics, New York University Grossman School of Medicine, New York City, USA

**Keywords:** refusal to bear weight, psoas muscle, hip pain, fever, psoas abscess

## Abstract

A psoas abscess is described as a collection of pus in the iliopsoas muscle compartment, which comprises the psoas and iliacus muscles located in the extraperitoneal space. It can be considered a primary abscess due to hematogenous or lymphatic seeding from a distant site, primarily occurring in children in tropical or developing countries. These primary infections are typically due to a single microorganism, most commonly, *Staphylococcus aureus*. Secondary spread develops due to the direct spread of infection of the psoas muscle from an adjacent structure (hip, vertebrae, gastrointestinal tract, aorta, genitourinary tract), developing due to trauma or instrumentation of the inguinal region, lumbar spine, or hip region. The secondary infections can be either mono- or polymicrobial and include enteric and anaerobic organisms. We present a case of psoas abscess in a five-year-old female who presented with a progressively worsening pain in the right hip for three days with refusal to bear weight and no history of trauma. Hip x-ray imaging yielded no abnormal results, but laboratory values prompted further investigation, leading to identifying a right psoas abscess via MRI with surrounding edema and enhancement. Imaging modality choice has proven to be instrumental in identifying psoas abscess and is key to achieving a diagnosis.

## Introduction

Psoas abscess forms a collection of pus in the psoas muscle [1]. The psoas muscle originates from the transverse processes of the vertebral column and runs along with the vertebral bodies of the 12th thoracic to the fifth lumbar vertebrae. Psoas abscesses are thought to arise from contiguous spread from adjacent structures, hematogenous spread from distant sites of infections, or trauma or instrumentation used in the inguinal, hip, or lumbar regions. They can be divided into primary or secondary based on the pathogenesis, although it may be difficult to differentiate them in some circumstances [2]. A primary psoas abscess occurs from hematogenous or lymphatic spread [3-5]. Risk factors for this finding include diabetes, vertebral osteomyelitis or discitis, IV drug abuse, HIV, inflammatory bowel disease, renal failure, and various forms of immunosuppression [1,3]. Trauma and hematoma formation can predispose patients to the development of a primary psoas abscess [4]. It has also been a finding due to complications of epidural anesthesia [6]. The primary psoas abscess is more commonly seen in children and young adults [3,4,7]. A secondary psoas abscess is caused by a direct spread from an adjacent structure. However, it is sometimes difficult to differentiate whether the adjacent structure is the cause of infection or the result of a psoas muscle abscess [8]. Risk factors for secondary abscesses include trauma and instrumentation in the inguinal, hip, or lumbar regions [8-11]. Examples of adjacent structures responsible for contiguous spread include the vertebral bodies, vertebral discs, hip joints in patients who have undergone hip arthroplasty, the gastrointestinal tract, nearby vascular structures such as the aorta, and the genitourinary tract [2,8]. The most common pathogen associated with a psoas abscess is *Staphylococcus aureus*, including methicillin-resistant *S. aureus* (MRSA), followed by *Streptococci* and *Escherichia coli*. Secondary psoas abscesses can be mono- or polymicrobial. The most common associated polymicrobial pathogens are enteric organisms. Patients with a psoas abscess may have symptoms that can mimic spinal arthritis, pain in the lower extremity, and abdominal pain. Psoas abscesses have also been described in the setting of appendicitis, colorectal cancer, ulcerative colitis, and after abdominal surgery [3,7,12]. Therefore, further investigation is ideal for a more accurate and supportive diagnosis.

## Case presentation

A five-year-old female child with no significant past medical history presented to the emergency department with atraumatic, progressively worsened right hip pain for three days associated with refusal to bear weight with vital signs remarkable for fever of 100.5°F, and on physical exam, with no swelling or deformity of the hip. However, she had pain on passive range of motion of the right hip, with resistance to active movement of the hip. On initial laboratory studies, she had leukocytosis with a WBC count of 16.17 x 10^3^/mcL, neutrophilia of 78% and elevated C-reactive protein level 40.7 mg/L, elevated erythrocyte sedimentation rate (ESR) of 43 mm/hr, and blood culture positive for *S. aureus*, as well as a normal x-ray of the hip and femur.

**Figure 1:**
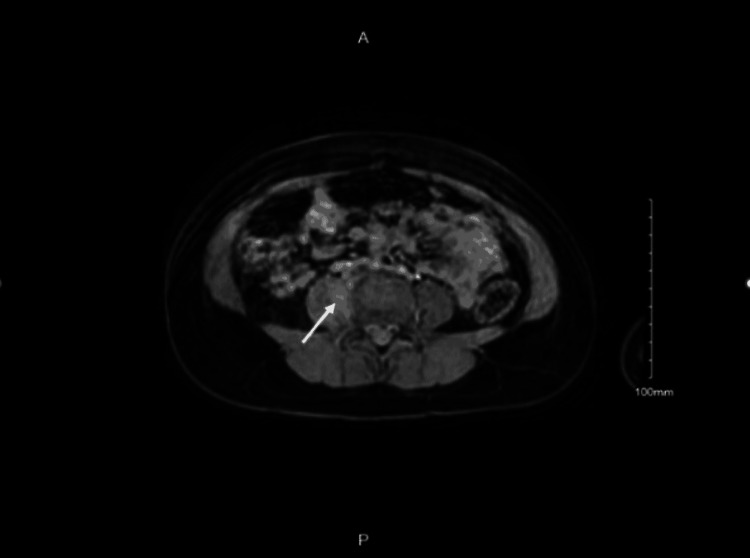
Abdominopelvic MRI showing a 5-mm right psoas abscess with surrounding edema and enhancement (white arrow)

This patient was transferred to a tertiary center for the pediatric orthopedic management of possible right hip septic arthritis. However, when evaluated with magnetic resonance imaging (MRI), this clinical suspicion was ruled out by the findings showing no evidence of septic arthritis or osteomyelitis, but rather a finding of right psoas muscle edema that warranted further evaluation with abdominopelvic MRI as shown in Figure 1. The latter found a 5-mm right psoas abscess with surrounding edema and enhancement.

The management of the patient included inpatient admission, appropriate analgesia with opioids and anti-inflammatory medications, as well as IV antibiotics followed by first-generation oral cephalosporin for a total of three weeks. The patient was discharged home when clinical improvement was observed, and she was able to ambulate without pain.

## Discussion

Psoas abscess is a rare diagnosis in children but that is commonly overlooked in the nonverbal pediatric age group [13,14]. In several pediatric case series, different age ranges have been reported. These included ages 4 to 14 years, 11 months to 13 years, and 18 months to 14 years [15-17]. Our patient did not fall in the common age range of patients with psoas abscess.

Seeing that no secondary source of infection was identified in our patient, her psoas abscess is likely of primary etiology, of which the pathogenesis is usually blurred or idiopathic. Several studies demonstrated that most pediatric psoas abscess cases are primary [16]. In one study, a history of specific trauma before the onset of symptoms was elicited in 3 out of 12 patients. There is a strong association of trauma with psoas abscess [17]. Other postulations for the cause of primary abscesses include suppuration of lymphadenitis and hematogenous spread from a skin infection. Sources implicated in causing the secondary cases included perinephric abscess, acute appendicitis, Pott’s disease, and sacroiliitis [15]. Epidemiologically, the literature search found the majority of pediatric cases in Africa and Asia as primary versus secondary in Europe and North America [18].

Presentations in the literature varied and drifted far from the classic presentation first reported in 1881, which comprised a triad of back pain, limp, and a fever. Our patient presented with an insidious onset of nonspecific symptoms including hip pain, limping, and inability to bear weight, which prompted the provisional diagnosis of septic arthritis. The past literature reported various clinical features including flank/back pain, vague abdominal pain, limp, fever, malaise, weight loss, and groin lump [18]. However, abdominal pain, fever, gait disturbances, psoitis, and painful abdominal mass were the most common clinical signs [16,17]. On the other hand, fever and back pain were identified in all patients investigated [16].

Regarding the microbiological diagnosis, cultures from most cases usually return positive, with the most common causative agent being *S. aureus* [15]. It accounts for 80%-90% of cases of primary psoas abscess in children. Along the same lines, our patient’s cultured sample grew methicillin-susceptible *S. aureus* (MSSA). MRSA is a concern in certain geographical regions where its prevalence is known to be higher [14].

As both septic arthritis and psoas abscess can present with limp and hip pain as well as systemic features like fever, misdiagnosis is common. Both may also present as referred pain in the thigh and back. However, abdominal pain may be more suggestive of a psoas abscess [13]. The diagnosis of septic arthritis is especially favored when patients meet the Kocher criteria for septic arthritis as seen in our patient who had 4/4 positive criteria (non-weight bearing, temperature >38.5, ESR >40 mm/hr, and WBC >12,000 cells/mm³). Some studies even described psoas abscess as a consequence of septic arthritis although others couldn’t recover this relation [19].

MRI was key in the diagnosis of this patient. This concurs with previous studies that highlighted the importance of imaging, particularly MRI. MRI is better than computed tomography (CT) due to better visualization of soft tissues. It clearly delineates the abscess wall and surrounding structures and establishes the diagnosis of a psoas abscess. Although ultrasound eliminates the risk of radiation, it poses the challenges of operator-dependent sensitivity as well as the difficulty of investigating the retroperitoneal area due to the presence of intestinal gas [13]. Although CT has been associated with false-negative results, it holds high sensitivity when combined with ultrasound and is particularly beneficial in management through guided drainage [18].

Several options of treatment are available for psoas abscess; treatment modalities include antibiotic therapy (empiric followed by targeted therapy), drainage (radiologically guided or surgery), and debridement. The initial empiric antimicrobial therapy is usually based on epidemiologic factors, including local sensitivities and resistance features [20,21]. The choice of antibiotic therapy and duration of treatment is uncertain, although some authors consider three to six weeks of therapy to be satisfactory after drainage [20]. It was ascertained that solitary antibiotic therapy is more effective if used in the early stages of abscess formation or for abscesses not exceeding 3 mm [15].

Surgical interventions are recommended for cases that fail less invasive management options, including antimicrobial therapy and radiologically guided drainage to decrease morbidity (iliacus abscess) [21]. Regarding surgical drainage, approaches include intraperitoneal, anterolateral, or posterior based on the abscess location [21]. In our case, the patient was initially started on vancomycin and then transitioned to cefazolin as culture results revealed MSSA growth. Additionally, the 5-mm collection detected on MRI supported the choice of management with antibiotics alone as it was too small for drainage. Subsequent blood cultures returned negative, which drove the decision of switching the patient to oral cephalexin for an additional three weeks.

## Conclusions

Psoas abscess is an uncommon presentation in both the adult and pediatric populations and can have a vague clinical presentation. This may lead to delays in diagnosis, resulting in high morbidity and mortality; hence, a high level of suspicion is required for diagnosis. One or more signs and symptoms such as groin pain, limping, fever, abdominal pain, and back pain may be considered as early signs of a psoas abscess. Painful hip, abdominal pain, fever, and gait disturbances are among the common presentations.

Though several studies have demonstrated the cause of psoas abscess in the pediatric population to be primary, it is important that clinicians look out for other risk factors in children such as trauma and compromised immunity as well as secondary etiologies. Prompt diagnosis from clinical presentation, laboratory findings, and imaging will help improve early management and reduce the risk of complications, mortality, and morbidity.
